# Site-specific assessment of spinal radiographic progression improves detection of TNF blocker-associated disease modification in axial spondyloarthritis: longitudinal observational data from the Swiss Clinical Quality Management Registry

**DOI:** 10.1186/s13075-023-03026-6

**Published:** 2023-03-13

**Authors:** Vjara Popova, Seraphina Kissling, Raphael Micheroli, René Bräm, Manouk de Hooge, Xenofon Baraliakos, Michael J. Nissen, Burkhard Möller, Pascale Exer, Michael Andor, Oliver Distler, Almut Scherer, Caroline Ospelt, Adrian Ciurea

**Affiliations:** 1 Department of Rheumatology, Zurich University Hospital, University of Zurich, Rämistrasse 100, CH-8091 Zurich, Switzerland; 2 Swiss Clinical Quality Management Foundation, Statistics Group, Zurich, Switzerland; 3 Swiss Ankylosing Spondylitis Association, Zurich, Switzerland; 4 VIB Inflammation Research Center, Ghent University, Ghent, Belgium; 5 Department of Rheumatology, Ghent University Hospital, Ghent, Belgium; 6 Rheumazentrum Ruhrgebiet Herne, Ruhr-University Bochum, Bochum, Germany; 7 Department of Rheumatology, University Hospital Geneva, Geneva, Switzerland; 8 Deparment of Rheumatology and Immunology, University Hospital Bern, Bern, Switzerland; 9 Praxis Rheuma-Basel, Basel, Switzerland; 10 Rheumatology Practice, Uster, Switzerland

**Keywords:** Axial spondyloarthritis, Ankylosing spondylitis, Tumour necrosis factor inhibitor, Radiographic progression

## Abstract

**Objectives:**

To analyse whether time-varying treatment with tumour necrosis factor inhibitors (TNFi) in radiographic axial spondyloarthritis (r-axSpA) has a differential impact on structural damage progression on different spinal segments (cervical versus lumbar spine).

**Methods:**

Patients with r-axSpA in the Swiss Clinical Quality Management cohort were included if cervical and lumbar radiographs were available at intervals of 2 years for a maximum of 10 years. Paired radiographs were scored by two calibrated readers according to the modified Stoke Ankylosing Spondylitis Spine Score (mSASSS). The relationship between TNFi use and progression in the cervical and the lumbar spine was analysed using generalised estimating equation models and adjustment for potential confounding. Radiographic progression per spinal segment was defined as an increase of ≥ 1 mSASSS unit or by the formation of ≥ 1 new syndesmophyte over 2 years.

**Results:**

Mean ± SD symptom duration was 13.8 ± 9.8 years. Mean ± SD mSASSS progression per radiographic interval was 0.41 ± 1.69 units in the cervical spine and 0.45 ± 1.45 units in the lumbar spine (*p* = 0.66). Prior use of TNFi significantly reduced the odds of progression in the cervical spine by 68% (OR 0.32, 95% CI 0.14–0.72), but not in the lumbar spine (OR 0.99, 95% CI 0.52–1.88). A more restricted inhibition of progression in the lumbar spine was confirmed after multiple imputation of missing covariate data (OR 0.43, 95% CI 0.24–0.77 and 0.85, 95% CI 0.51–1.41, for the cervical and lumbar spine, respectively). It was also confirmed with progression defined as formation of ≥ 1 syndesmophyte (OR 0.31, 95% CI 0.12–0.80 versus OR 0.56, 95% CI 0.26–1.24 for the cervical and lumbar spine, respectively).

**Conclusion:**

Disease modification by treatment with TNFi seems to more profoundly affect the cervical spine in this r-axSpA population with longstanding disease. Site-specific analysis of spinal progression might, therefore, improve detection of disease modification in clinical trials in axSpA.

**Supplementary Information:**

The online version contains supplementary material available at 10.1186/s13075-023-03026-6.

## Background

Impairments in physical function in ankylosing spondylitis (AS) are determined by both disease activity and spinal mobility [1]. The latter is independently associated with spinal inflammation as well as with structural damage [2]. The disease-modifying capacity of tumour necrosis factor inhibitors (TNFi)—approved in AS and axial spondyloarthritis (axSpA) for their effect on inflammation—has long been questioned [3]. Several recent studies evaluating the impact of time-varying treatment with TNFi on spinal radiographic progression in patients with AS (meanwhile referred to as radiographic (r)-axSpA [4]) have consistently found slowing of osteoproliferative changes [5]. Their study design, including adjustment for time-varying potential confounders, suggest causality, although the definitive demonstration of the latter might be more difficult to confirm [5].

Axial disease in axSpA usually starts in the sacroiliac joints and later involves the spine [6–8]. There is evidence that syndesmophyte formation progresses from caudal to cranial [9–11]. Radiographic progression at different time-points might, therefore, differ in the cervical versus the lumbar spine. Distinct progression rates in spinal segments might also be due to differences in mechanical load and biomechanical forces, given that mechanical strain might enhance new bone formation in spondyloarthritis [12]. Finally, overlapping degenerative changes might differentially affect spinal segments [13, 14] and affect evaluation of progression of lesions associated with axSpA. We thought to evaluate whether the demonstrated impact of time-varying treatment with TNFi on spinal progression as assessed by validated methods might differ in the cervical versus the lumbar spine by re-analysing data from a large national cohort of patients with r-axSpA.

## Methods

### Study population

We took advantage of a large national observational registry of patients diagnosed with axSpA by their treating rheumatologist, the Swiss Clinical Quality Management (SCQM) axSpA cohort [15]. Patients were included in the current study if they fulfilled the Assessment of SpondyloArthritis international Society (ASAS) classification criteria for axSpA [16] and the radiographic item of the modified New York criteria [17] and if they had at least two sets of lateral radiographs of the cervical and the lumbar spine with an interval of 2 years ± 1 year. Clinical assessments were performed according to the recommendations of the ASAS [18]. The study was approved by the Ethics Committee of the Canton of Zurich (KEK-ZH-Nr. 2014–0439 and BASEC-Nr. 2022–00,272). All patients provided written informed consent prior to recruitment into SCQM.

### Assessment of radiographic progression

The study represents a statistical re-analysis of scoring data of the SCQM registry published previously [15]. We used our original scoring of spinal radiographs according to the modified Stoke Ankylosing Spondylitis Spinal Score (mSASSS) [19] but divided for the current analysis the total mSASSS (range 0–72) into the cervical mSASSS and the lumbar mSASSS (range 0–36 for each segment). As reported [15], all radiographs per patient were scored by two trained readers (MdH, XB) with knowledge of chronology but blinded to all clinical data. Both readers have extensive experience in scoring of spinal imaging in axSpA and were also involved in studies evaluating potentially overlapping degenerative disease [14, 20]. Radiographs were excluded if > 3 vertebral corners (VC) in both the cervical and lumbar spine were missing. An adaptation algorithm was used to impute individual missing VCs [21] as detailed in the supplementary appendix. An independent adjudicator (AC) scored all X-rays from a patient, if an absolute difference in mSASSS status scores of at least 5 units was detected between the primary readers in at least one radiograph set. Averaged scores per vertebral corner were used and, in case of adjudication, the score of the primary reader closest to the adjudicator.

Radiographic progression for the cervical and lumbar spine was defined as an increase in cervical mSASSS and in lumbar mSASSS of ≥ 1 unit over an interval of 2 years in the respective spinal segment. We alternatively defined radiographic progression as an increase in cervical or lumbar mSASSS of ≥ 2 units over 2 years in sensitivity analyses (supplementary appendix). Moreover, we assessed the percentage of patients with formation of at least one syndesmophyte in the cervical spine and the lumbar spine, respectively. Syndesmophytes were only counted if both readers agreed upon their presence.

### Statistical analyses

The relationship between treatment (TNFi and/or NSAIDs) and radiographic progression of ≥ 1 mSASSS unit per spinal segment was analysed using generalised estimating equations (GEE) with an “exchangeable” correlation structure.

Based on the results of our previous analysis and findings in other cohorts [5], any treatment with TNFi prior to the radiographic interval was used as the variable representing TNFi treatment (with the majority of patients being treated with TNFi for at least 2 years [15]). Time-varying information on regular NSAIDs treatment was available at start of each interval as “yes/no,” without information on whether the agent used was a traditional NSAID or a coxib. The models were further adjusted for sex, symptom duration, human leucocyte antigen B27 (HLA-B27) status, smoking status, presence of peripheral arthritis, body mass index (BMI) categories, length of the radiographic interval, and baseline radiographic damage (either mSASSS at start of the interval or the presence of syndesmophytes in any spinal segment). The models were further adjusted for the number of physical activity sessions per week as a proxy for mechanical strain on the spine. This variable combined information available from a patient questionnaire on type of exercise (axSpA gymnastics in groups or at home, training in fitness centres or other) and its frequency (1–2x/week; 3–4x/week; 5–7x/week) without data on duration of the respective exercise sessions. Time-varying disease activity parameters (e.g. C-reactive protein (CRP)) were not included in the models, as these variables were shown to mediate the effect of TNFi on radiographic progression [15]. The issue of confounding by indication was addressed by adjusting for the ASDAS-CRP value before the start of TNFi treatment in an additional model. ASDAS-CRP at inclusion in the SCQM cohort was considered in this model for patients not treated with TNFi. To investigate the issue of missing values, the GEE models were also fitted using multiple imputation of missing covariate data (supplementary appendix). The R statistical software was used for all analyses.

## Results

### Unadjusted analyses

**Fig. 1:**
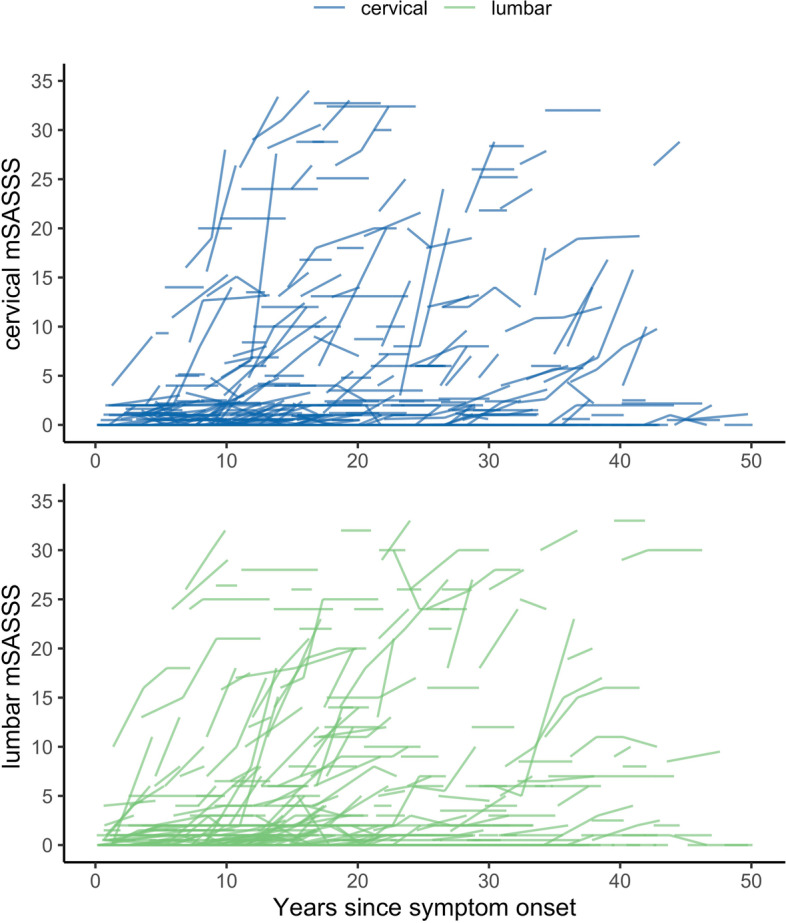
Modified Stoke Ankylosing Spondylitis Spinal Score (mSASSS) stratified by spinal segments (range 0–36) and shown for individual patients plotted as a function of duration since symptom onset. Progression in the cervical spine in blue (upper part of the figure), progression in the lumbar spine in green (lower part of the figure)

Demographic and clinical characteristics of 433 patients with r-axSpA and at least two sets of lateral radiographs of the cervical and the lumbar spine with an interval of 2 years ± 1 year are shown at start of first radiographic interval in Table 1. Reliability of mSASSS scoring for this population has already been presented [15] and was considered “good” (ICC 0.85). It was slightly better for the lumbar spine scoring in comparison to the cervical spine scoring (ICC 0.90, 95% CI 0.85–0.92 versus ICC 0.73, 95% CI 0.65–0.78, respectively). Mean (SD) total progression per radiographic interval was 0.86 (2.53) mSASSS units, with no difference observed between the cervical and the lumbar segment of the spine: 0.41 (1.69) units versus 0.45 (1.45) units, respectively (Welch two-sample *t*-test *p*-value 0.66; confidence interval (CI) of the difference in means: − 0.22 to 0.14). We did not observe a difference with regard to the appearance of new syndesmophytes at the cervical vs. the lumbar level: mean (SD) new cervical syndesmophyte number 0.16 (0.66) vs. 0.17 (0.58) for the lumbar spine (CI for the difference in means − 0.09 to 0.06, *p* = 0.75). The cervical and lumbar mSASSS is depicted separately for individual patients as a function of symptom duration in Fig. 1. Relevant progression in the cervical spine was only visible from the end of the first decade onwards, while it started earlier in the lumbar spine.

**Table 1:** Baseline characteristics at first radiograph

Parameter	All patients		Patients with complete covariate data	
	***N***		***N***	
Male sex, *N* (%)	433	285 (65.8)	297	197 (66.3)
HLA-B27 positive, *N* (%)	392	316 (80.6)	297	240 (80.8)
Age, years	433	40.3 (11.0)	297	39.5 (10.6)
Symptom duration, years	425	13.8 (9.8)	297	13.5 (9.4)
BASDAI	369	4.2 (2.3)	286	4.3 (2.3)
ASDAS-CRP	351	2.8 (1.1)	276	2.9 (1.1)
CRP (mg/l), median (IQR)	365	8.0 (3.0; 11.0)	278	8.0 (4.0; 12.0)
Elevated CRP, *N* (%)	364	147 (40.4)	277	116 (41.9)
BASFI	373	3.1 (2.6)	288	3.1 (2.5)
BASMI	375	2.2 (2.0)	285	2.2 (2.5)
mSASSS median (IQR)	433	1.0 (0.0; 6.0)	297	1.0 (0.0; 6.0)
Mean (SD)		6.6 (12.5)		6.6 (12.6)
Syndesmophytes present, *N* (%)	433	148 (34.2)	297	98 (33.0)
EQ-5D	370	65.1 (21.6)	284	64.5 (21.5)
Current peripheral arthritis, *N* (%)	378	108 (28.6)	289	82 (28.4)
Current enthesitis, *N* (%)	381	207 (54.3)	288	171 (59.4)
BMI 25–30, *N* (%)	373	110 (29.5)	287	84 (29.3)
BMI > 30, *N* (%)	373	58 (15.6)	287	42 (14.6)
On NSAID treatment, *N* (%)	341	286 (83.9)	277	236 (85.2)
On TNFi treatment, *N* (%)	433	163 (37.6)	297	96 (32.3)
Ever TNFi treatment, *N* (%)	433	186 (43.0)	297	116 (39.1)
Years of TNFi treatment in treated patients	163	2.1 (1.7)	96	2.0 (1.6)
Current smokers, *N* (%)	366	140 (38.2)	285	104 (36.5)
Number exercise sessions per week	366	2.0 (0.0; 4.0)	286	2.0 (0.0; 4.0)
Patients with different number of radiographic intervals, *N* (%)	433	100	297	100
1 interval	294	67.9	182	61.3
2 intervals	92	21.2	76	25.6
3 intervals	35	8.1	28	9.4
4 intervals	11	2.5	10	3.4
5 intervals	1	0.2	1	0.3

Except where indicated otherwise, values are the mean (SD). *ASDAS-CRP* Ankylosing Spondylitis Disease Activity Score using C-reactive protein levels, *BASDAI* Bath Ankylosing Spondylitis Disease Activity Index, *BASFI* Bath Ankylosing Spondylitis Functional Index, *BASMI* Bath Ankylosing Spondylitis Metrology Index, *BMI* Body mass index, *CRP* C-reactive protein (CRP) levels, *EQ-5D* EuroQol 5-domains, *HLA-B27* Human leucocyte antigen B27, *mSASSS* modified Stoke Ankylosing Spondylitis Spinal Score, *NSAID* Nonsteroidal anti-inflammatory drug, *TNFi* Tumour necrosis factor inhibitor

### Adjusted longitudinal analyses

**Fig. 2:**
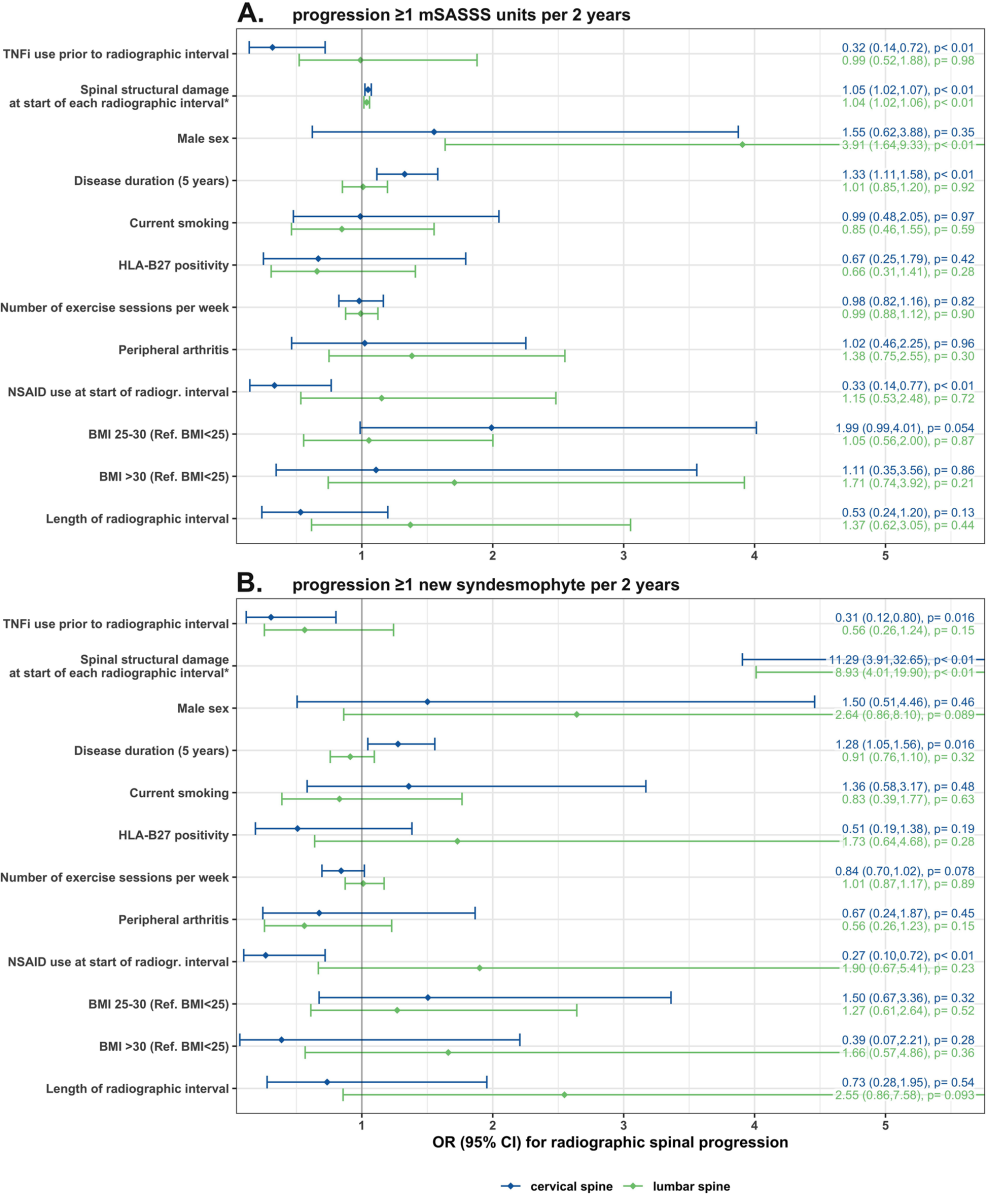
Multivariable analysis of 616 radiographic intervals from 432 patients for the identification of factors associated with spinal radiographic progression in the cervical spine (blue) and the lumbar spine (green). Progression was defined as **A** an increase in ≥ 1 mSASSS unit per spinal segment in 2 years and **B** as the formation of at least one new syndesmophyte per spinal segment in 2 years. BMI, body mass index; HLA-B27, human leucocyte antigen B27; mSASSS, modified Stoke Ankylosing Spondylitis Spinal Score; NSAIDs, nonsteroidal anti-inflammatory drugs; Ref, reference; TNFi, tumour necrosis factor inhibitor. Asterisk symbol (*) indicates the following: mSASSS at start of each 2-year radiographic interval in **A** and presence of syndesmophytes at start of each 2-year radiographic interval (yes vs no) in **B**

**Fig. 3:**
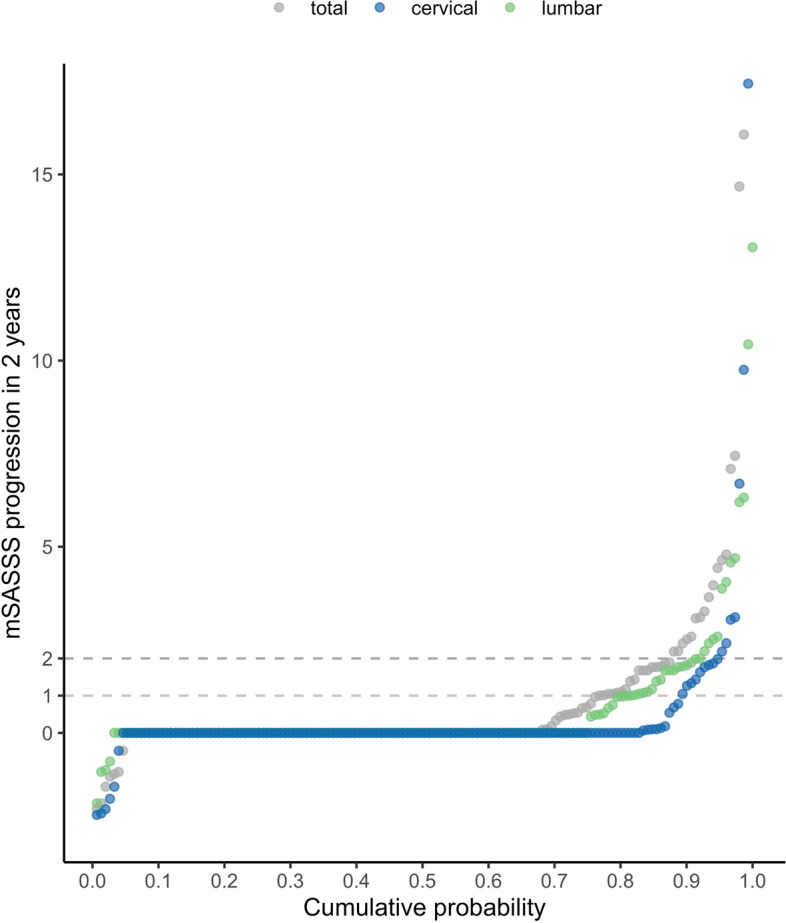
Spinal radiographic progression in patients with high risk of progression (patients already treated with TNFi, but not reaching an ASDAS ≤ 2.1 at the beginning of each radiographic interval). Cumulative probability plot of 2-year progression in the modified Stoke Ankylosing Spondylitis Spinal Score (mSASSS; range 0–72), illustrating the change in total (cervical + lumbar) mSASSS values from baseline of each individual radiographic interval to 2 years (grey). The mSASSS values for the cervical and lumbar spinal segments (range 0–36) are shown in blue and green, respectively. Radiographic progression was defined as an increase in total mSASSS of ≥ 2 units in 2 years and an increase of ≥ 1 unit if only the cervical or lumbar segments of the spine were considered (dotted lines)

Results of adjusted longitudinal analyses to assess factors impacting on spinal radiographic progression are shown separately for the cervical and the lumbar spine in Fig. 2. Treatment with TNFi before the start of the radiographic interval was associated with a much lower odds ratio for progression by ≥ 1 mSASSS unit in the cervical spine (OR 0.32, 95% CI 0.14–0.72) in comparison to the 50% reduction in progression when the cervical and the lumbar spine were analysed together in our previous publication [15], due to the fact that progression was not significantly retarded in the lumbar spine (OR 0.99, 95% CI 0.52–1.88) (Fig. 2A). A higher reduction of the odds of progression in the cervical spine compared to the lumbar spine was also observed with progression defined as the formation of ≥ 1 syndesmophyte (OR 0.31, 95% CI 0.12–0.80 for the cervical spine versus OR 0.58, 95% CI 0.26–1.24 for the lumbar spine) (Fig. 2B). The results were confirmed in several sensitivity analyses: (a) with progression defined as an increase in ≥ 2 mSASSS units per spinal segment (Supplementary Table S1), (b) after multiple imputation of missing covariate data (Table 2A), and (c) after additional adjustment for disease activity as assessed by the ASDAS at start of treatment to address the potential issue of confounding by indication (Table 2B). Lower mSASSS progression in the cervical versus the lumbar spine upon TNFi treatment is illustrated in a cumulative probability plot for patients with high risk of further progression (TNFi treated patients not reaching an ASDAS-CRP ≤ 2.1 (ASDAS low disease activity) at start of the radiographic interval) in Fig. 3. In contrast, progression was almost completely inhibited in patients treated with TNFi reaching remission (ASDAS-CRP ≤ 1.3) before the radiographic interval for both the cervical and the lumbar segment of the spine (Supplementary Fig. 1).

**Table 2:** Sensitivity analyses performed after multiple imputation of missing covariate data. Models with and without ASDAS at treatment start to address the potential issue of confounding by indication

		A. **Model without ASDAS**			B. **Model with ASDAS**		
**Spinal segment**	**Variable**	**OR**	**95% CI**	***P* value**	**OR**	**95% CI**	***P* value**
Cervical spine	TNFi use before radiographic interval yes/no	0.43	0.24; 0.77	0.004	0.42	0.23; 0.76	0.004
	Total mSASSS at start of each radiogr. interval	1.05	1.03; 1.07	< 0.001	1.05	1.03; 1.07	< 0.001
	Male sex	1.29	0.68; 2.43	0.44	1.28	0.68; 2.42	0.45
	Symptom duration (5 years)	1.22	1.07; 1.40	0.004	1.22	1.07; 1.40	0.003
	Current smoking	1.24	0.69; 2.23	0.47	1.23	0.68; 2.22	0.50
	HLA-B27	0.72	0.33; 1.58	0.42	0.72	0.33; 1.59	0.42
	Number of exercise sessions per week	0.99	0.87; 1.12	0.86	0.99	0.87; 1.12	0.85
	Peripheral arthritis	1.18	0.63; 2.23	0.60	1.17	0.62; 2.22	0.63
	NSAID use at start of each radiographic interval	0.52	0.26; 1.02	0.06	0.51	0.26; 1.01	0.054
	BMI 25–30 (reference: BMI < 25)	1.46	0.81; 2.63	0.21	1.45	0.80; 2.62	0.22
	BMI > 30 (reference: BMI < 25)	1.08	0.47; 2.50	0.85	1.07	0.46; 2.49	0.88
	Length of radiographic interval	0.87	0.47; 1.61	0.67	0.88	0.47; 1.62	0.67
	ASDAS at start of TNFi and ASDAS at inclusion for non-treated patients				1.05	0.75; 1.46	0.79
Lumbar spine	TNFi use before radiographic interval yes/no	0.85	0.51; 1.41	0.53	0.72	0.43; 1.21	0.22
	Total mSASSS at start of each radiogr. interval	1.04	1.02; 1.06	< 0.001	1.04	1.03; 1.06	< 0.001
	Male sex	2.85	1.51; 5.35	0.001	2.83	1.51; 5.31	0.001
	Symptom duration (5 years)	1.08	0.95; 1.22	0.27	1.09	0.96; 1.24	0.19
	Current smoking	0.78	0.47; 1.29	0.33	0.73	0.44; 1.22	0.22
	HLA-B27	0.68	0.36; 1.26	0.22	0.69	0.37; 1.28	0.24
	Number of exercise sessions per week	1.01	0.91; 1.13	0.81	1.01	0.91; 1.12	089
	Peripheral arthritis	1.13	0.65; 1.97	0.66	1.05	0.59; 1.85	0.87
	NSAID use at start of each radiographic interval	1.25	0.67; 2.34	0.48	1.18	0.63; 2.21	0.60
	BMI 25–30 (reference: BMI < 25)	1.42	0.81; 2.49	0.23	1.37	0.77; 2.44	0.28
	BMI > 30 (reference: BMI < 25)	2.06	1.06; 4.01	0.03	1.90	0.99; 3.67	0.06
	Length of radiographic interval	1.55	0.82; 2.93	0.18	1.57	0.82; 3.02	0.17
	ASDAS at start of TNFi and ASDAS at inclusion for non-treated patients				1.31	0.94; 1.82	0.11

Progression defined as an increase in mSASSS of at least 1 unit in 2 years. *ASDAS*, Ankylosing Spondylitis Disease Activity Score; *BMI*, body mass index; *HLA-B27*, human leucocyte antigen B27; *mSASSS* modified Stoke Ankylosing Spondylitis Spinal Score, *NSAID* Nonsteroidal anti-inflammatory drug, *TNFi *Tumour necrosis factor inhibitor

### The impact of additional factors on spinal radiographic progression

Baseline radiographic damage was the most important predictor of further mSASSS progression in both spinal segments (Fig. 2 and Table 2). The number of exercise sessions per week—as a proxy for mechanical strain—had no consistent impact on mSASSS progression (Fig. 2 and Table 2). The regular use of NSAIDs was, comparably to the use of TNFi, also associated with reduced progression exclusively in the cervical spine (OR 0.33, 95% CI 0.14–0.77 and OR 1.15, 95% CI 0.53–2.48 for the cervical and the lumbar spine, respectively, Fig. 2) with statistical significance lost, however, in the sensitivity analyses performed (Table 2). To better understand the impact of sex on radiographic progression, we excluded baseline damage from the main model (Table 3). Male sex was significantly associated with radiographic progression in both the cervical and the lumbar spine in this model, an effect that was partly concealed if the baseline damage was considered in the original model. The size of the effect induced by treatment with TNFi on progression was only minimally affected by this change (Table 3). Current smoking was not associated with radiographic progression neither in the cervical spine nor in the lumbar spine.

**Table 3:** Analysing spinal progression in the cervical versus the lumbar spine after excluding baseline structural damage from the models to better depict the impact of sex

		**Progression ≥ 1 mSASSS units per 2 years**			**Progression ≥ 1 new syndesmophyte per 2 years**		
**Spinal segment**	**Variable**	**OR**	**95% CI**	***P* value**	**OR**	**95% CI**	***P* value**
Cervical spine	TNFi use before radiographic interval yes/no	0.41	0.19; 0.87	0.02	0.37	0.13; 1.03	0.06
	Male sex	2.68	1.09; 6.62	0.03	3.66	1.25; 10.7	0.02
	Symptom duration (5 years)	1.47	1.25; 1.73	< 0.001	1.49	1.23; 1.80	< 0.001
	Current smoking	1.05	0.53; 2.08	0.88	1.23	0.57; 2.66	0.60
	HLA-B27	0.52	0.22; 1.22	0.13	0.39	0.15; 1.02	0.05
	Number of exercise sessions per week	0.97	0.84; 1.13	0.72	0.84	0.71; 0.99	0.04
	Peripheral arthritis	0.81	0.37; 1.76	0.59	0.59	0.22; 1.60	0.30
	NSAID use at start of each radiographic interval	0.47	0.21; 1.04	0.06	0.40	0.14; 1.12	0.08
	BMI 25–30 (reference: BMI < 25)	1.79	0.91; 3.52	0.09	1.35	0.62; 2.96	0.45
	BMI > 30 (reference: BMI < 25)	0.92	0.29; 2.98	0.90	0.43	0.08; 2.24	0.32
	Length of radiographic interval	0.52	0.23; 1.16	0.11	0.72	0.26; 1.96	0.52
Lumbar spine	TNFi use before radiographic interval yes/no	1.10	0.60; 2.03	0.76	0.73	0.35; 1.53	0.41
	Male sex	5.63	2.36; 13.5	< 0.001	5.52	1.85; 16.4	0.002
	Symptom duration (5 years)	1.12	0.97; 1.30	0.13	1.11	0.94; 1.31	0.21
	Current smoking	0.86	0.48; 1.55	0.61	0.69	0.33; 1.45	0.33
	HLA-B27	0.56	0.27; 1.15	0.12	1.17	0.42; 3.27	0.77
	Number of exercise sessions per week	0.98	0.87; 1.10	0.76	0.94	0.83; 1.08	0.39
	Peripheral arthritis	1.18	0.65; 2.13	0.58	0.55	0.26; 1.17	0.12
	NSAID use at start of each radiographic interval	1.39	0.68; 2.85	0.37	2.64	0.94; 7.42	0.07
	BMI 25–30 (reference: BMI < 25)	1.01	0.54; 1.88	0.99	1.07	0.52; 2.22	0.85
	BMI > 30 (Reference: BMI < 25)	1.44	0.65; 3.20	0.37	1.46	0.55; 3.88	0.44
	Length of radiographic interval	1.30	0.58; 2.90	0.53	2.22	0.77; 6.38	0.14

*ASDAS *Ankylosing Spondylitis Disease Activity Score, *BMI *Body mass index, *HLA-B27 *Human leucocyte antigen B27, *mSASSS *Modified Stoke Ankylosing Spondylitis Spinal Score, *NSAID *Nonsteroidal anti-inflammatory drug, *TNFi *Tumour necrosis factor inhibitor

## Discussion

Our re-analysis of the longitudinal assessment of spinal structural damage by region in a large cohort of patients with r-axSpA reveals that the retardative effect of TNFi treatment on radiographic progression [5] is not equally distributed between the spinal segments. A much greater effect can be detected in the cervical spine than the one found for the whole spine in our registry [15], explained by a smaller magnitude of the impact—not reaching statistical significance—in the lumbar spine. This result was found with progression defined as an increase in mSASSS of at least 1 or 2 units per spinal segment, as well as the formation of at least 1 syndesmophyte. It was confirmed in several sensitivity analyses: after multiple imputation of missing covariate data, after the addition of disease activity as assessed by the ASDAS at treatment start; and after exclusion of baseline structural damage.

The mSASSS remains the most validated and widely used method to assess spinal radiographic progression in axSpA, despite progress achieved in the area of imaging [22]. The standardised clinical and radiographic assessments at regular intervals and statistical methods that take into account not only potential confounders but also the within-patient correlation of structural damage represent important strengths of our study.

How can the finding of a comparable crude radiographic progression in the cervical and the lumbar spine over 2 years be explained in light of a more profound drug-induced inhibition of progression in the cervical spine over the same period? A higher natural progression rate in the cervical spine in patients with comparable mean symptom duration would be compatible with both findings. The fact that we found that most structural damage progression in the first 5 years of disease seems to be confined to the lumbar spine would be reconcilable with a more important cervical progression at later time-points and with previous studies having suggested disease progression from caudal to cranial [9–11]. It is important to note, that it remains unknown, whether the progression rates demonstrated in early AS studies really represent “natural” progression, given the fact that the disease-modifying effect of treatment with NSAIDs remains controversial [23, 24]. This issue is discussed in more detail below, all the more we found a site-specific impact of treatment with NSAIDs comparable to the one of TNFi.

Several, mutually not exclusive hypotheses can be put forward to explain a differential inhibition of progression in the cervical versus the lumbar spine. The first hypothesis is related to the fact that structural changes seem to start in the lumbar spine. Inhibition of progression might not be possible any more if certain reparative changes have already been initiated, and this might occur at an earlier time-point in the lumbar spine. Magnetic resonance studies have demonstrated that syndesmophyte formation is more likely to occur at VCs in which inflammation has been replaced by fatty degeneration, than at VCs with persistent inflammation [25]. The fact that structural damage seems to start in the caudal spine would imply that in the first few years after start of symptoms, inhibition of progression would only be detectable in the lumbar spine. Given the long mean symptom duration in our cohort, the number of patients with early disease was too low to allow testing this assumption. In line with this argumentation, inhibition of progression should be detected at all spinal levels if TNFi are initiated early on and sustained remission is achieved. Indeed, almost no progression could be detected in patients reaching an ASDAS < 1.3 before a next radiographic interval at both cervical and lumbar level in our study. The regional difference in inhibition of progression was most clearly depicted in patients with persisting high disease activity despite bDMARD treatment. A second hypothesis involves the presence of degenerative spinal disease that might interfere with the assessment of axSpA-induced osteoproliferative changes. Indeed, degenerative changes overlap with axSpA-associated lesions even in early disease and most frequently involve the more distal aspects of the spine [13, 14]. However, it has been shown that trained readers are able to distinguish between axSpA-associated versus degenerative lesions [14, 20]. Our primary readers were involved in these studies, rendering this hypothesis less probable, though not absolutely impossible. Thirdly, mechanical strain was shown to be able to enhance new bone formation [12]. Biomechanical forces are larger at the level of the lumbar spine and might lead to enhanced progression in the caudal region of the spine and potentially counteract pharmacological inhibitory effects. However, there were no clues for progression being more important in the lumbar spine during late disease. Moreover, the number of physical exercise sessions per week, introduced as a proxy for physical strain in our investigation, did not significantly impact on the results. Finally, site-specific developmental differences might be involved. Joint-specific anatomical diversity has been demonstrated for synovial fibroblasts and for cartilage with regard to the expression of homeobox (HOX) family genes [26, 27], which are crucial for the embryonic development of limb and vertebrae. Imprinted developmental differences could therefore also control site-specific activation of axSpA-relevant pathways in the entheses along the different regions of the spine.

Irrespective of the mechanism(s) leading to differential regional inhibition of progression as delineated above, the applicability of our findings is manifold. The clinical relevance of retardation of radiographic progression in axSpA upon treatment with TNFi has been questioned [5]. Indeed, progression is not linear in the individual patient. Moreover, it only affects a proportion of patients in a given interval of 2 years. Our data suggesting that inhibition of progression might be restricted to certain spinal segments further challenges its clinical relevance. However, the inhibitory effect on cervical structural damage is of such magnitude (68% odds reduction of radiographic progression for TNFi) that it seems of utmost clinical relevance for the patients concerned, particularly for rapidly progressing patients. The more important potential to detect inhibition of spinal radiographic in the cervical spine might also have an influence on the demonstration of the disease-modifying effect of other drug classes. The issue of inhibition of radiographic progression by nonsteroidal anti-rheumatic drugs (NSAIDs) remains, as already mentioned, controversial. Two randomised controlled trials investigating the effects of on-demand use versus continuous use of different classes of NSAIDs on spinal progression in AS reached opposite conclusions [23, 24]. In our main models, treatment with NSAIDs reached a retardative impact on radiographic progression in the cervical spine that was comparable in size to treatment with TNFi at this spinal segment, while no significant effect of NSAIDs was detected in our original analysis of total mSASSS. The fact that we could not calculate a NSAIDs intake score as recommended by the ASAS [28] and no information was available on the type of NSAID used (traditional NSAIDs or coxibs) represent major limitations of our current analyses. Time-varying treatment with NSAIDs was only available as “yes/no” at start of each interval and this information was included as such in our models, assuming that rheumatologists mainly considered a “yes” when the patient used NSAIDs on a regular basis and that this was continued in the following radiographic interval. Whether the putative symptom duration-dependent differential inhibitory effect on progression in the cervical versus the lumbar spine also applies to treatment with NSAIDs and might explain the previously contrary results obtained in trials assessing the impact of NSAIDs on progression, therefore, remains unclear. Site-specific re-analysis of previously performed trials, as well as newly designed trials, seem warranted to confirm our findings. Our findings could also be of importance when comparing the disease-modifying capacities of different classes of biologic or targeted-synthetic disease modifying drugs in axSpA in head-to-head clinical trials. Site-specific assessment might improve detection of potential differences in progression, particularly as the expected differences might be rather small [28]. As demonstration of causality is difficult to achieve in an observational context [5], only comparative head-to-head trials will be able to provide a definitive answer to the conundrum whether disease modification through inhibition of osteoproliferation is possible in axSpA, provided that a difference in progression between patients treated with different drugs can indeed be detected.

Our study confirms a more important spinal radiographic progression in men in comparison to women in both spinal segments. Current smoking was not associated with spinal progression in neither the cervical spine nor the lumbar spine. An effect of smoking on spinal osteoproliferation was found in some but not in all studies that have considered this lifestyle factor in the respective analyses [15, 29]. The reason for these discrepancies might involve the different populations analysed and the fact that smoking might confound the amplifying impact of mechanical stress (e.g. job type) on the potentiating effects of inflammation on radiographic progression [30, 31].

## Conclusions

Our study points to differences in site-specificity of radiographic progression in AS, most probably in dependence on symptom duration of the population assessed. The magnitude of the retardative impact of TNFi on progression at the level of specific spinal segments seems greater than previously demonstrated, with respective implications for early treatment of rapid progressors. The potential to detect inhibition of spinal radiographic more readily with a site-specific approach might have an influence on the demonstration of the disease-modifying effect of other drug classes, particularly in head-to-head clinical trials, as the expected differences in progression within a time-frame of 2 years are rather small [32].

## Supplementary Information

**Additional file 1: Supplementary Methods.** Adaptation algorithm for spinal radiographic scores. Imputation of missing covariate data.  **Supplementary Table 1.** Multivariable analysis for identification of factors associated with radiographic progression defined as an increase of **≥ 2** mSASSS units per 2 years in the cervical and in the lumbar spine. **Supplementary Figure 1.** Cumulative probability plot of 2-year progression in the modified Stoke Ankylosing Spondylitis Spinal Score (mSASSS) by spinal segments, illustrating the change in mSASSS values in patients already treated with TNFi at start of the respective interval, stratified by the ASDAS cut-off level reached at baseline.

## Data Availability

Restrictions apply to the availability of these data. Data is owned by a third party, the Swiss Clinical Quality Management in Rheumatic Diseases (SCQM) foundation. Data may be obtained after approval and permission from the license holder (SCQM). Contact information for data request: scqm@hin.ch.

## Abbreviations

AS: Ankylosing spondylitis
ASAS: Assessment in SpondyloArthritis international Society
ASDAS: Ankylosing Spondylitis Disease Activity Score
AxSpA: Axial spondyloarthritis
BASDAI: Bath Ankylosing Spondylitis Activity Index
BASFI: Bath Ankylosing Spondylitis Functional Index
BASMI: Bath Ankylosing Spondylitis Mobility Index
BMI: Body mass index
CRP: C-reactive protein
EQ-5D: European quality of life 5 domains questionnaire
GEE: Generalised estimating equations
HLA-B27: Human leucocyte antigen B27
mSASSS: Modified Stoke Ankylosing Spondylitis Spinal Score
nr-axSpA: Nonradiographic axial spondyloarthritis
NSAID: Nonsteroidal anti-inflammatory drug
r-axSpA: Radiographic axial spondyloarthritis
SCQM: Swiss Clinical Quality Management
TNFi: Tumour necrosis factor inhibitor
VC: Vertebral corner
