# Supplementary material for: Site-specific assessment of spinal radiographic progression improves detection of TNF blocker-associated disease modification in axial spondyloarthritis: longitudinal observational data from the Swiss Clinical Quality Management Registry
Source: Arthritis Res Ther. 2023 Mar 13;25:40. doi: 10.1186/s13075-023-03026-6 (PMC10009926; doi:10.1186/s13075-023-03026-6)
Supplement: Supplementary file 1 — Additional file 1: Supplementary Methods. Adaptation algorithm for spinal radiographic scores. Imputation of missing covariate data. Supplementary Table 1. Multivariable analysis for identification of factors associated with radiographic progression defined as an increase of ≥ 2 mSASSS units per 2 years in the cervical and in the lumbar spine. Supplementary Figure 1. Cumulative probability plot of 2-year progression in the modified Stoke Ankylosing Spondylitis Spinal Score (mSASSS) by spinal segments, illustrating the change in mSASSS values in patients already treated with TNFi at start of the respective interval, stratified by the ASDAS cut-off level reached at baseline. [file 13075_2023_3026_MOESM1_ESM.docx]

**Supplementary Appendix**

**Supplementary Methods**

*Adaptation algorithm for spinal radiographic scores*

An adaptation algorithm was used to impute individual missing verterbral corners (VCs) in the scoring of spinal cervical and lumbar radiographs according to the modified Stoke Ankylosing Spondylitis Spinal Score (mSASSS): A missing value for a VC was replaced with the value of the previous observation. Secondly, the mean progression score at either the cervical or the lumbar level per patient was calculated. This segmental progression score was added to the imputed value, keeping in mind that the score per VC could never exceed a score of 3. A score of 0 was assumed for missing previous time-point(s) if at a later time-point a score of 0 was found in the same VC. If the baseline score of a VC was missing, the same procedure was applied, subtracting the mean segment progression from the score of year 2 for a particular patient. If a value of this VC was also missing at year 2, then the average of the other available VCs from this spinal segment at baseline was used to replace the missing VC(s). Imputation of missing VC scores was performed for 487/16’632 VCs (2.9%) for scorer 1 and 644/16’680 VCs (3.9%) for scorer 2 for the serial radiographs of 297 patients included in the main adjusted model.

*Imputation of missing covariate data*

The GEE models were fitted using multiple imputation of missing covariate data. Out of 616 intervals, 212 (35%) had at least one missing value in one of the 12 variables used in the GEE. The proportion of missing values varied from 0% to 19%. An individual GEE was fitted for each of the imputed dataset. Pooling of model estimates was performed according to Rubin’s rules. A total of 30 imputed datasets were created. The ASDAS was derived by passive imputation. Predictive mean matching was used for continuous variables and logistic regression for binary variables. Convergence of imputations was assessed by visual inspection of the mean and variance changes by iteration and dataset.

**Supplementary Table 1.** Multivariable analysis for identification of factors associated with radiographic progression defined as an increase of **≥2** mSASSS units per 2 years in the cervical and in the lumbar spine.

|  | **Cervical spine** | | | **Lumbar spine** | | |
| --- | --- | --- | --- | --- | --- | --- |
| **Variable** | **OR** | **95% CI** | **P value** | **OR** | **95% CI** | **P value** |
| TNFi use before radiographic interval yes/no | 0.34 | 0.12; 0.95 | 0.04 | 0.97 | 0.47; 2.03 | 0.94 |
| mSASSS at start of each radiographic interval | 1.05 | 1.02; 1.08 | <0.001 | 1.04 | 1.02; 1.07 | <0.001 |
| Male sex | 2.15 | 0.62; 7.43 | 0.23 | 4.75 | 1.34; 16.8 | 0.02 |
| Symptom duration (5 years) | 1.28 | 1.04; 1.57 | 0.02 | 0.89 | 0.72; 1.10 | 0.28 |
| Current smoking | 1.51 | 0.64; 3.57 | 0.34 | 0.54 | 0.23; 1.25 | 0.15 |
| HLA-B27 | 0.51 | 0.24; 2.71 | 0.73 | 0.97 | 0.33; 2.88 | 0.96 |
| Number of exercise sessions per week | 0.93 | 0.78; 1.12 | 0.45 | 1.00 | 0.86; 1.16 | 0.98 |
| Peripheral arthritis | 1.32 | 0.55; 3.17 | 0.54 | 0.91 | 0.43; 1.92 | 0.80 |
| NSAID use at start of each radiographic interval | 0.48 | 0.17; 1.39 | 0.18 | 1.09 | 0.44; 2.72 | 0.85 |
| BMI 25-30 (Reference: BMI <25) | 2.05 | 0.92; 4.56 | 0.08 | 1.07 | 0.48; 2.38 | 0.86 |
| BMI >30 (Reference: BMI <25) | 0.52 | 0.07; 4.02 | 0.53 | 1.85 | 0.75; 4.57 | 0.18 |
| Length of radiographic interval | 0.55 | 0.23; 1.33 | 0.19 | 2.09 | 0.85; 5.15 | 0.11 |

Analysis performed in 399 intervals from 297 patients. BMI = Body Mass Index; HLA-B27 = human leucocyte antigen B27; mSASSS = modified Stoke Ankylosing Spondylitis Spine Score; NSAID = Nonsteroidal anti-inflammatory drug; TNFi = Tumour necrosis factor inhibitor.


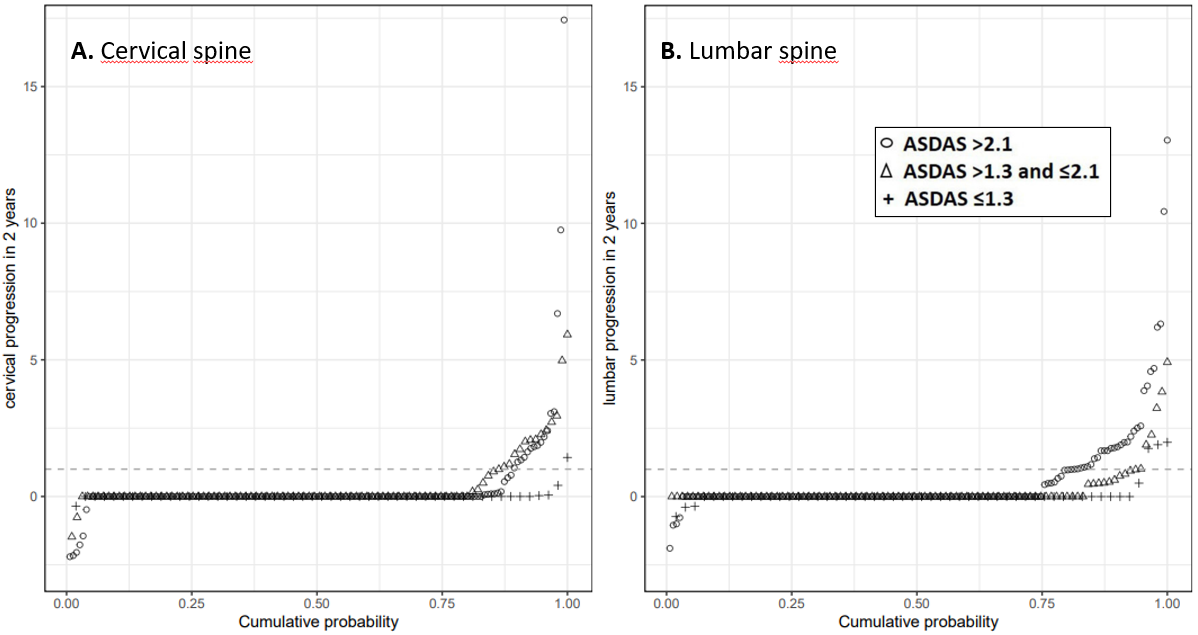


**Supplementary Figure 1**. Cumulative probability plot of 2-year progression in the modified Stoke Ankylosing Spondylitis Spinal Score (mSASSS) by spinal segments (**A.** cervical spine; **B.** lumbar spine); illustrating the change in mSASSS values (range 0-36 per spinal segment) from baseline of each radiographic interval to 2 years in patients already treated with TNFi at start of the respective interval, stratified by the ASDAS cut-off level reached at the beginning of each radiographic level. Radiographic progression was defined as an increase in mSASSS of ≥1 unit in 2 years (dotted line). ASDAS, Ankylosing Spondylitis Disease Activity Score.
